# Cholera Outbreaks in Zimbabwe: An In-Depth Analysis of Drivers, Constraints and Reimagining the Use of Medicinal Plants

**DOI:** 10.1155/jotm/1981991

**Published:** 2024-11-21

**Authors:** Jerikias Marumure, Monde A. Nyila

**Affiliations:** Department of Life and Consumer Sciences, College of Agriculture and Environmental Sciences, Science Campus, University of South Africa, Florida, Johannesburg 1710, South Africa

**Keywords:** cholera control challenges, cholera outbreaks, epidemiology, medicinal plants, potential treatment options, *Vibrio cholerae*

## Abstract

Cholera, an intestinal infection caused by *Vibrio cholerae*, poses a severe threat to public health, particularly in developing countries. This narrative review discusses drivers for cholera outbreaks, challenges and viable alternatives, in Zimbabwe. A literature search was conducted using electronic databases notably ScienceDirect, Google Scholar and PubMed, as well as thesis and conference papers. Evidence indicates that the epidemiology, as well as risk factors, includes (1) extreme droughts; (2) political meddling in health issues and water supply; (3) natural disasters; (4) migration; (5) problems with water and sanitation; and (6) the endemic nature of the causative agent as well as its development of drug resistance. Reliable supply of clean water and proper sanitation and hygiene as the main key to prevention is emphasised. The use of antibiotics and vaccines for therapy, as well as the use of medicinal plants in traditional medicine, is discussed. *Kirkia acuminata* and *Ziziphus mucronata* root and stem bark infusions or decoctions were revealed to be the most common folklore treatments for cholera in rural communities. The potential of medicinal plants as anti-*Vibrio cholerae* remedies based on their positive antibacterial assays, and mechanism of action is also presented. Finally, the development of innovative anti-*Vibrio cholerae* therapeutics based on natural leads and compounds and adapted for use in resource-constrained cholera-prone areas is viewed as a potential option, to complement cholera prevention and treatment, particularly in resource-limited endemic areas.

## 1. Introduction

Cholera, a gastrointestinal infection caused by *Vibrio cholerae*, continues to present a significant risk to public health [1]. However, effective control of cholera necessitates the implementation of various components, such as surveillance, treatment, water and sanitation measures, and vaccinations [2, 3] which collectively render the disease highly manageable and preventive. Even though the disease can be easily treated and prevented, it is still poorly managed in numerous epidemic and endemic regions, especially in countries with high human poverty indices [4]. For example, Zimbabwe presently has a population in which 42% of individuals reside in dire poverty [5], and the country has been grappling with recurring cholera outbreaks. Nevertheless, in light of the acknowledged risk factors linked to the disease, numerous questions persist that necessitate additional exploration. Why can't Zimbabwe prevent cholera outbreaks from occurring? Why is Zimbabwe unable to contain cholera outbreaks as they occur? Amidst the ongoing enquiries, the most catastrophic outbreak occurred in 2008, with the case fatality ratio (CFR) escalating to 20% [6, 7]. The most recent outbreak was recorded in February 2023 [6], and there has been a continuous increase in the number of cases. In 2024 alone, there have been a total of 35,000 documented cases and 600 deaths [8] Moreover, in many of the outbreaks, the CFR exceeds the acceptable level of 1% set by the World Health Organisation (WHO) [2, 8].

**Figure 1:**
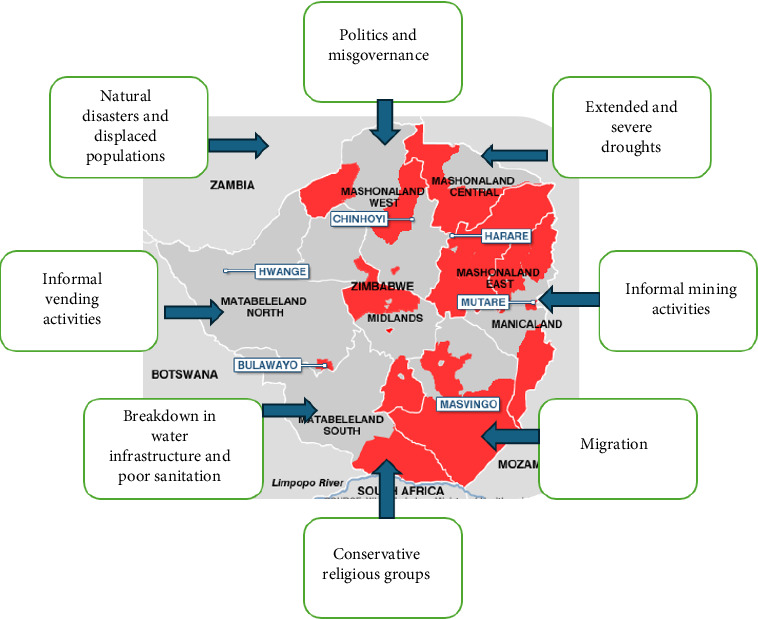
An overview of typical drivers for the repeated occurrences of cholera outbreaks in Zimbabwe (map of Zimbabwe adapted from BBC News [13]).

Based on recent and previous epidemics, several studies, including anecdotal evidence, have revealed an array of underlying issues or drivers for Zimbabwe's periodic cholera outbreaks [6, 7, 9–12]. An overview of typical drivers for the repeated occurrences of cholera outbreaks in Zimbabwe is presented in Figure 1. These drivers are not exclusive to Zimbabwe; they are also present in other southern African countries that have recently faced cholera outbreaks [14–16]. For example, the cholera outbreak in Zambia was attributed to the presence of contaminated water sources, inadequate sanitation and hygiene practices, and insufficient healthcare facilities [14]. Similarly, there is anecdotal evidence, indicating that the current cholera outbreak in South Africa was a result of malfunctioning and noncompliant wastewater treatment facilities, mismanagement, inadequate investment and misappropriation of funds [17].

To address the multidisciplinary character of cholera control and management, this paper also suggests incorporating natural products, especially in resource-poor endemic areas that are more susceptible to cholera. Natural products such as plants have been viewed as potential remedies for a wide range of infections [18, 19]. Management of diseases using medicinal plants is not a new approach. In most developing countries, 70%–95% of people rely on medicinal plants for basic health care [20, 21]. A few studies, including surveys and reviews, as well as anecdotal evidence, have revealed various medicinal plants that local communities utilise to manage diarrhoea and cholera [22–24]. However, scientific evidence to support the vibriocidal and therapeutic properties of the reported medicinal plants is still limited. Nevertheless, the anti-*Vibrio cholerae* activity of various medicinal plant extracts, including isolated phytochemicals, from elsewhere is documented in several studies [25–27]. Furthermore, while not mutually exclusive, different mechanisms of action of plant extracts or isolated phytochemicals on *V. cholerae* and related bacteria have been revealed by a number of studies, including: (1) cell membrane disruption [25, 28, 29], (2) suppression of quorum sensing (QS) [25, 30], (3) inhibition of virulence factor production [31, 32], (4) inhibition of biofilm formation [32, 33], (5) inhibition of protein synthesis and function [27, 34] and (6) synergy or potentiation of commonly used antibiotics [35, 36].

The current review intends to address the drivers of cholera in Zimbabwe while also aiming to answer the following questions: Why can't Zimbabwe prevent cholera outbreaks? Why is Zimbabwe struggling to contain cholera epidemics when they occur? The challenges that contribute to the recurrence of cholera outbreaks, as well as the difficulties in managing the epidemics, will be discussed. The possibility of returning to conventional cholera treatment based on traditional medicine and culture is considered. More importantly, the potential and efficacy of medicinal plants as anti-*Vibrio cholerae* treatments is investigated. Finally, the review's findings will provide new insights into how cholera might be controlled, particularly in low-income countries, as future research directions to fill the existing knowledge gaps are highlighted.

## 2. Materials and Methods

The current study used a literature study approach, retrieving information from English-language academic electronic databases using a Boolean search method (e.g., Google Scholar, Web of Science, Scopus and ScienceDirect). In Boolean search techniques, terms or search strings are combined with operators and modifiers (AND, OR and NOT) to narrow or broaden the search. This approach enabled the retrieval of the most literature related to the study objectives, but may have excluded other papers published in non-English language. Both American and English variants of terms were used in the search process. The beginning of the search was not specified, but it was to last until the present (2024). Typical search strings used include the following: *‘drivers of cholera in low income settings*' *AND/OR ‘cholera treatment' AND/OR ‘vibriocidal medicinal plants' AND/OR ‘antidiarrhoeal plants' AND/OR ‘challenges in cholera treatment', AND/OR ‘potential cholera treatment alternatives', AND/OR ‘mechanisms of medicinal plants in cholera treatment'* and other related search strings.

It should be noted that a fully systematic quantitative review using bibliometric and meta-analysis approaches was outside the scope of this work.

## 3. Results

### 3.1. Drivers Contributing to the Recurring Cholera Epidemics in Zimbabwe

**Figure 2:**
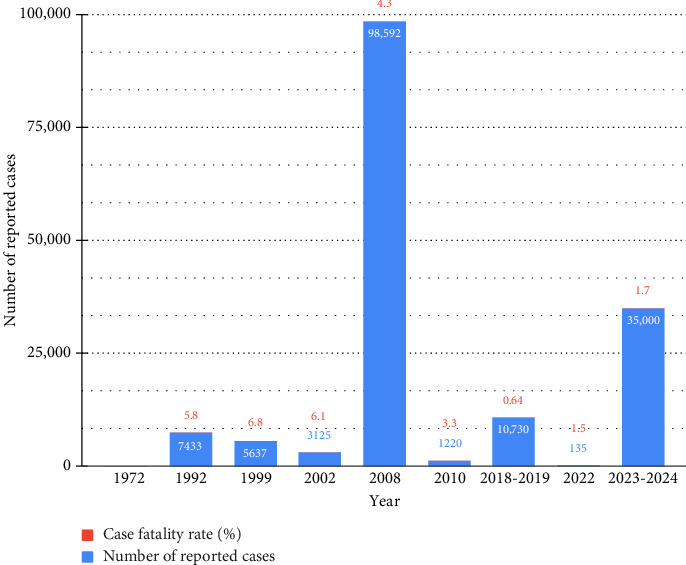
Cholera outbreaks in Zimbabwe, with complete statistics missing for 1972.

Zimbabwe has seen cholera outbreaks since 1972 (Figure 2). Until now, cholera has been endemic; the most current outbreak began in February 2023, and cases are steadily growing, with 35,000 reported cases and 600 deaths in 2024 alone [8]. The disease is easily treatable and preventable; however, various challenges and seemingly overwhelming drivers in Zimbabwe's current situation allow the disease to remain a significant public health issue in the country.

#### 3.1.1. Breakdown in Water Infrastructure and Poor Sanitation

**Figure 3:**
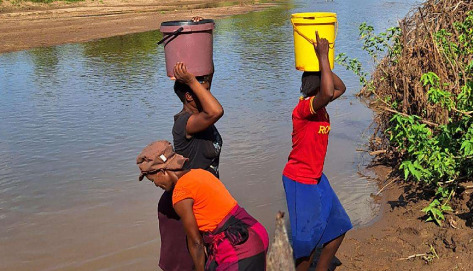
Villagers fetching water for drinking in the river in Mbire District (adapted from Médecins Sans Frontières [8]). Photographer: MSF/GRACE MAVHEZHA.

Zimbabwe's recurring challenges from the past outbreaks [37] have paved the way for recent and ongoing cholera outbreaks [6]. In both rural and urban areas, vulnerabilities in water, sanitation and hygiene (WASH) infrastructure have heightened the risk of transmission [8]. In Harare, contaminated water from boreholes and wells was suspected to be the source of the outbreaks [38], as drinking water from some boreholes has been found to be contaminated by sewage leaking from broken pipes [39]. Recently, shortage of potable water was linked to loss of mega volumes of treated water everyday due to old water pipes bursting, leaving residents with not enough water, hence resorting to alternative sources which may be unsafe [40]. Moreso, most townships in Harare and other cities have been reported to go for weeks, or even months, without a regular supply of water from the City Council [41]. Furthermore, anecdotal evidence has revealed that most Harare residents have stopped drinking water from the tap, terming it ‘green' and they have resorted to drinking water sourced from boreholes and shallow wells, which could be contaminated also. Moreover, the situation is compounded by raw sewage becoming a common sight in townships, uncollected garbage and unsafe hygiene practices mostly in major cities and towns. In the rural areas, recent findings indicate that many boreholes have become inoperable, leaving people with no choice except to consume unsafe water from the nearby streams (Figure 3). This water source is often shared with both wild and domestic animals [8].

The dilapidated water and sewage system, in Zimbabwe, have been a persistent problem for almost 2 decades now. According to [42] Muronzi, the current sewage system is part of its inherited colonial infrastructure and has been overwhelmed as the population grew from about 3.7 million in 1960 to 17 million today. As Zimbabwe is experiencing or recovering from a debilitating social and economic crisis, which began in the last 2 decades, it is failing to invest in maintaining critical infrastructure for water and sewerage reticulation, resulting in frequent bursts and collapse of the systems [43]. Even with assistance from nongovernmental organisations (NGOs) and international agencies (e.g., United Nations International Children's Emergency Fund [UNICEF]), the country is failing to meet the required US$8,047,500 to provide enough essential WASH services, among other services, to combat the current and ongoing outbreak [6, 9]. Along with this background, for months now, Zimbabwe has been battling to stem the spread of deadly cholera in its cities and villages because the country simply lacks clean water and proper sewage disposal [41].

#### 3.1.2. Natural Disasters and Displaced Populations

Several studies have demonstrated how natural disasters (floods, earthquakes and tropical cyclones) cause significant population displacement and transmission of diseases. References [44–46] found that displaced populations are often susceptible to a range of infectious diseases, such as cholera. This is because natural disasters have the potential to contaminate or destroy water sources and sanitary infrastructure, leading to the rapid spread of the disease [47]. Although natural disasters in Zimbabwe are rare, there have been a few studies that have established a connection between the occurrence of cholera and these disasters. For example, in 2014, there was an outbreak of diarrhoeal sickness among 1500 people who were homeless and relocated due to the floods caused by the Tokwe-Mukosi Dam partial collapse [48]. Consequently, the absence of safe sources of water necessitated people to depend on stagnant water, which presented a potential hazard to their health [49]. Similarly, Cyclone Idai, which impacted the Southern Hemisphere between 4 and 21 March 2019, resulted in the loss of lives and displacement of numerous families, particularly in Zimbabwe, Mozambique and Malawi [50]. In Zimbabwe, specifically in the Chimanimani and Chipinge districts, a total of 51,000 individuals were rendered homeless. This led to the occurrence of diarrhoeal diseases, including cholera, in the campsites and collective centres due to the lack of access to clean water and proper sanitation facilities [51].

#### 3.1.3. Extended and Severe Droughts

A few studies have established a connection between droughts and cholera, particularly in sub-Saharan Africa [52–55]. Charnley, Kelman and Murray [54] discovered that prolonged and severe droughts have resulted in several factors that increase the risk of cholera, including malnutrition, insufficient access to WASH and population displacement. Although droughts are not explicitly identified as risk factors for cholera in the WHO guidelines, a study by Rieckmann et al. [55] demonstrated that there were 276 cholera outbreaks during 118 droughts between 1990 and 2010 in 41 sub-Saharan countries. Thus, droughts play a significant role in contributing to the occurrence of cholera. Furthermore, the same authors contended that droughts could have an adverse effect on population hygiene, resulting in a heightened susceptibility to diarrhoeal outbreaks, such as cholera. Zimbabwe has experienced numerous droughts in the past. Significant and prolonged periods of drought have been observed in the following years: 1972–1973 [56], 1991–1992, 1994–1995, 2002–2003, 2015–2016, 2018–2019 [57, 58] and in 2023–2024 [39, 59]. The droughts have led to water scarcity, reduced agricultural productivity and occurrences of food insecurity, thus leading to economic downturns [58] and a potential vulnerability to cholera transmission. Significantly, previous cholera outbreaks in Zimbabwe (Figure 1) have co-occurred with the periods mentioned above of drought. For example, the current cholera outbreak in 2023–2024 has occurred at the same time as the government's declaration of a national disaster due to drought [36].

#### 3.1.4. Politics and Misgovernance

Several studies have demonstrated that cholera epidemics in Zimbabwe have occurred due to political causes [60–62]. In 2000, Zimbabwe had a notable shift in its political environment. An influential opposition party arose, resulting in persistent disputes. The ruling party, leveraging its powers at the national level, obstructed the opposition, which often held sway over local government [63]. Subsequently, the delivery of services, such as providing clean drinking water and sewage disposal, was impacted, leading to a rise in regular outbreaks of cholera. Following that, the government has faced consistent scrutiny from the public populace about cholera outbreaks [10]. For instance, the 2008–2009 cholera epidemic, which lasted for 10 months, was highly linked to political manipulation [64]. Notably, the outbreak was attributed to human rights breaches and the politicisation of water, health care, aid and information. The outbreak occurred shortly after two violent rounds of presidential elections [12]. Furthermore, in 2023, Zimbabwe held its elections and was seen to be back at its familiar crossroads, with cholera outbreaks occurring after disputed elections [6]. Again, the outbreaks pointed to the government's lack of will or ability or both to stem the occurrences by providing fresh water [41]. Moreover, the cholera outbreak in Harare and other cities has been linked to the growing vulnerability of the urban poor, which is caused by their forced displacement and fuelled by conflicts over economically and politically important urban areas [65].

#### 3.1.5. Migration

Migrants have also been linked to cholera transmission [66]. Cholera outbreak in Zimbabwe in the late 1992 was connected to an influx of migrants escaping cholera-stricken Mozambique. Similarly, anecdotal evidence also pointed to migration as one of the causes of the Zimbabwean disastrous epidemic of 2008–2009, which also expanded to neighbouring Zambia and South Africa. For the recent 2023–2024 cholera outbreak, no migration from other countries was reported as the main driver for the outbreak. However, such information sometimes may not be made public, and some diarrhoeal cases that are not reported especially in the rural areas could be actually cholera cases. However, movement of people within the borders was linked to the spread of the outbreak. For example, the first patient was reported in Chegutu on 23 February 2023, and the numbers significantly rose as the disease spread to all geographical corners of the country [6]. Furthermore, there was a steep increase during the holidays as people travelled and gathered to celebrate with their extended families, giving the disease new opportunities to spread [67]. Another key risk factor for cholera in Zimbabwe is the increase in rural–urban migration, mainly in search of economic opportunities. As a result, this further strains the already strained water and sanitation infrastructure in most urban areas [38]. For example, the 2018 cholera epidemic, Glen view in Harare, an active informal trading area where people come from across the city and the rest of the country to trade was the epicentre of the outbreak [38].

#### 3.1.6. Other Drivers for Cholera Recurrence

##### 3.1.6.1. Informal Mining Activities

Informal mining has a historical association with the spread of cholera [68]. The mining communities in Zimbabwe have experienced a significant increase in cholera cases during the recent outbreak [69]. According to reports from WHO, artisanal miners obtain water from polluted sources such as abandoned mining pits and rivers nearby, which serves as a favourable environment for the spread of cholera [69]. In Shamva district, located in Mashonaland Central province, there were around 2850 reported cases of cholera and 94 deaths, accounting for 19.5% of the total cases in the country. The main factors contributing to the transmission included open defecation and inadequate sanitation facilities [8].

### 3.2. Challenges

Cholera, while treatable and manageable, can present many challenges that may hinder the process of treating, managing and preventing the disease under particular conditions. These issues encompass a scarcity of vaccinations to adequately serve the entire population, the emergence of multidrug-resistant strains of *Vibrio cholerae* and various other obstacles.

#### 3.2.1. Emergence of Multidrug-Resistant *Vibrio cholerae*

In recent decades, Zimbabwe has suffered substantial outbreaks of antibiotic-resistant *Vibrio cholerae* strains [70, 71], impeding efficient and timely cholera control. Notably, *Vibrio cholerae* O1 serotype Ogawa isolates from Zimbabwe's 2018–2019 cholera outbreak displayed resistance to practically all antibiotics available at the time, including ciprofloxacin, tetracycline and ceftriaxone [72]. To address the outbreak, the patient management prescription was changed from the less expensive and more widely available ciprofloxacin to the more expensive and effective azithromycin, which was not available in health facilities [37, 43]. This led to increased healthcare expenses, mortality and morbidity [71]. According to reports from the cholera outbreak in nearby Malawi, the strain is still endemic [73]. Moreover, Zimbabwe, a country that frequently has cholera epidemics, must always be ready to face such challenges due to the observed progressive growth in bacterial resistance to antimicrobial drugs [74].

#### 3.2.2. Vaccination

The combination of cholera vaccinations with other preventive measures has been demonstrated to be critical components of disease prevention and control, particularly in cholera-endemic areas [8, 75, 76]. Early introduction of vaccines during an outbreak has been shown to offer 79% protection against cholera [76]. According to the same perspective, a number of studies reached the same conclusion about the possibility that mass immunisation could have mitigated the severity of the 2008 cholera outbreak in Zimbabwe [64, 77, 78]. Likewise, prompt immunisation might have contained the 2018 outbreak [64]. Moreover, the 1-month vaccine campaign for the recent 2023–2024 outbreak solely targeted 2.3 million people, living in high-risk districts. A mere 2,303,248 vaccination doses were obtained and administered [79], against the whole population of Zimbabwe which stands at 17,010,832, as of Monday, 24 June 2024 (https://www.worldometers.info/world-population/zimbabwe-population/). This low vaccination coverage has considerably contributed to the continuous spread of cholera in the country [6], as have previous epidemics. All of this can be attributable to the fact that there is a global shortage of oral cholera vaccines (OVCs) today, and Zimbabwe does not have enough doses to reach a larger population [8]. Furthermore, Euvichol-Plus, one of the presently common OVCs globally [75], was utilised in the most recent outbreak despite having a low and transient efficacy—two doses only offering 3 years of cholera protection. Furthermore, other constraints such as the necessity for a cold supply chain, production costs and complex vaccine distribution logistics reduce the widespread use of cholera vaccines in low-income countries like Zimbabwe [80].

### 3.3. Traditional Treatment of Cholera in Zimbabwe

The ongoing cholera outbreak in Zimbabwe demands a multifaceted strategy, which includes promoting traditional medicine. Improving infrastructure for clean water and acceptable sanitation during an ongoing outbreak may be difficult, especially if the country is still experiencing or recovering from an economic crisis [64]. As a result, this study considers the use of medicinal plants in cholera management, as plants have been identified as potential remedies for a variety of diseases [18, 19]. More importantly, this could be beneficial, especially in resource-limited endemic areas. Several studies, including surveys, reviews and anecdotal evidence, have discovered medicinal plants employed in folkloric treatment of cholera and other similar diarrhoeal diseases (Table 1). For example, *Ziziphus mucronata* root extracts [81, 92] and *Kirkia acuminata* bark extracts [23, 84, 85] are used to treat diarrhoeal diseases such as cholera, diarrhoea and dysentery. Similarly, decoctions or powdered bark of *Crossopteryx febrifuga* Benth. and *Sclerocarya birrea* Hochst. are mixed with meals or blown anally into the rectum [85]. More importantly, the usage of selected plant species as vibriocidal agents in traditional medicine is backed by documented data on antibacterial activity and phytochemical analysis (Table 1). However, Zimbabwe has yet to standardise and authorise the use of these therapeutic plant species in modern treatment. Furthermore, although several medicinal plants have shown efficacy in combating cholera or have been traditionally employed for cholera therapy [24, 93], it is important to note that they should not be relied upon as a complete substitute for mainstream cholera treatment or preventive methods.

### 3.4. Mechanism of Action of Plant-Based Medicines in Cholera Treatment

Several studies have demonstrated the effectiveness of plant extracts and isolated phytochemicals in suppressing the growth of *Vibrio cholerae* [25, 32, 94]. These activities, along with the well-documented folkloric use of certain plant species in managing cholera (Table 1), have sparked renewed efforts to incorporate them into the battle against cholera in Zimbabwe. Tables 2 and 3 present a summary, although not exhaustive, of the potential mechanisms that medicinal plants may utilise in the treatment of cholera, in both traditional and modern medicine.

#### 3.4.1. Disruption of Cell Membrane

Several studies have documented the impact of crude plant extracts and pure isolated phytochemicals on bacterial membranes [25, 113–115]. For example, in the study conducted by Tagousop et al. [29], purified flavonoid glycosides isolated from *Graptophyllum glandulosum* Turrill could trigger cell lysis by disrupting the cytoplasmic membrane of multidrug-resistant *Vibrio cholerae* strains. Membrane disruption encompasses the formation of pores, leakage of cellular contents, modifications in electrical charge and polarity, increased permeability, changes in fluidity and displacement of membrane proteins (Table 2). Plant compounds, such as galloyl catechins, gallotannin-1,2,3,4,5-penta-O-galloyl-*β*-D-glucose and some terpenoids and phytosterols, have been found to embed themselves into membranes, resulting in the lipid bilayer to becoming more fluid, exposing the membrane to damage [95, 116]. In some studies, catechin has been found to increase the generation of reactive oxygen species (ROS) within bacterial cells, leading to membrane permeabilisation by oxidative damage [100]. Furthermore, certain plant compounds can degrade or inhibit bacterial cell wall formation, leading to potential damage to the plasma membrane [34, 117]. Ultimately, all these changes and effects can culminate in the breakdown of cell integrity [98, 116, 117].

#### 3.4.2. Inhibition of Protein Synthesis and Function

The antibacterial activity of phytochemicals like tannins, flavonoids and terpenoids due to them interacting with bacterial proteins inhibiting their synthesis and function is documented (Table 2). According to Choudhary et al. [75], a number of saponins have been shown to penetrate the cytoplasmic membrane and then inhibit the normal synthesis of DNA and proteins that are required for bacterial growth. The effects of such phytochemicals are suggested to be at the molecular level rather than only physical damage. Another study [27] revealed that morin (3,5,7,2′,4′-pentahydroxyflavone), a flavonoid, was able to bind to major cell division protein of *V. cholerae* (VcFtsA) with a Kd of 4.68 ± 0.4 *μ*M, inhibiting the protein's polymerisation by 72% ± 7% at 25 *μ*M concentration, and in turn affected the cell division in *V. cholerae*. In similar studies, catechin and tannins isolated from *Uncaria gambir* Roxb. through molecular docking demonstrated their ability to bind to Muramidase B (MurB) enzyme, which is responsible for peptidoglycan biosynthesis in *Enterococcus faecalis*. The interaction might inhibit the action of the MurB enzyme, hence preventing bacterial cell wall formation [34, 114].

#### 3.4.3. Synergy Between Phytochemicals or With Commonly Used Antibiotics

**Figure 4:**
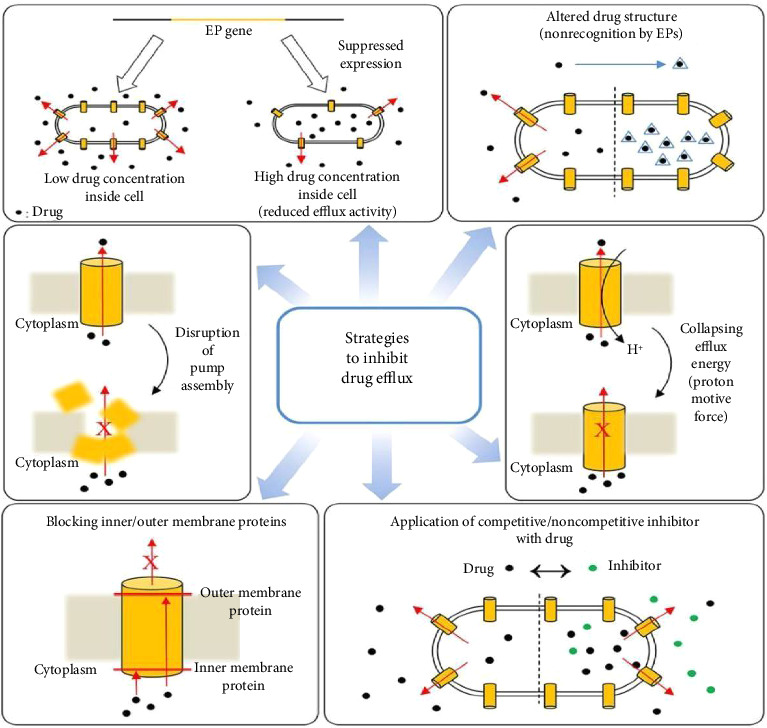
Strategies to inhibit drug efflux (adapted from Shriram et al. [120]).

Several studies have documented antibacterial activity of phytochemical–phytochemical or phytochemical–antibiotic combinations [35, 102, 118]. Wei et al. [96] indicated that the antibacterial activity of saponins can be significantly enhanced when used in combination rather than individually. More importantly, utilising the plant compounds in combination therapy has been shown to potentiate the antibiotics that would otherwise be losing their potency (Table 3). Costa, Pereira da Silva and Araújo [101] reported the ability of saponin-rich fractions to enhance the effect of gentamicin and norfloxacin against *Escherichia coli* and *Pseudomonas aeruginosa*. The ability of some phytochemicals to potentiate antibiotics is based on their ability to increase transport of antibiotics into bacterial cells [119] and to act as efflux pump inhibitors (EPIs) [120, 121]. For example, the synergistic co-action of the *Sapindus mukorossi* L. saponins and nitrofurantoin (NFT) resulted in 75% reduction in the activity of the cells as compared to 35% lower (after 8 h) when NFT was used alone on *Pseudomonas aeruginosa* NFT3 strain. The synergistic effect was due to membrane relaxation caused by saponins, which facilitates the bioavailability of NFT on the bacteria and increases its effectiveness as a biocidal agent [119]. The presence of efflux pumps has been discovered in most human pathogens, including in *Vibrio cholerae* [122]. By obstructing the efflux pump, these compounds enable antibiotics to enter and stay in the cell and exert their therapeutic effects [123]. Various mechanisms on how drug efflux is inhibited are documented (Figure 4). Several studies have discovered bioactive compounds that have the potential to act as EPIs [120]. For example, 8,8-bis(dihydroconiferyl)-diferulate from the bark of *Hypericum roeperianum* has been revealed to inhibit efflux pumps (e.g., the proton–ATPase pumps) in a wide range of multidrug-resistant bacteria [124]. Additionally, terpenoids and phytosterols have been reported to disrupt proteins embedded in the membrane, including efflux proteins [125].

#### 3.4.4. Inhibition of Biofilm Formation

Plant compounds have been found to possess the ability to combat biofilm formation. The mechanisms involved include the following: (1) suppression or downregulation of genes responsible for biofilm formation, (2) reduction in the production and secretion of the biofilm matrix and (3) the disruption of already formed biofilms (Table 3). For example, quercetin and naringenin displayed the ability to reduce the expression of biofilm-associated genes (*gbp*A, *vps*A, *rbm*A and *mba*A) in *Chromobacterium violaceum* and *Vibrio cholerae*. In addition, the same compounds exhibited antiadhesion and anti-invasion properties [26]. Furthermore, the genes (*aph*A or *aph*B) responsible for biofilm formation in toxigenic *Vibrio cholerae* were found to be downregulated by *Holarrhena antidysenterica*, *Elephantopus scaber* and *Centella asiatica* methanol extracts [107]. Similarly, Jantapaso et al. [25] revealed the ability of flavonoids isolated from *Nephelium lappaceum* L. cv. Rong Rian peel water extracts to significantly downregulate other genes involved in biofilm formation in *Vibrio cholerae* (*vps*L, *vps*R, *bap*1 and *rbm*A). More importantly, inhibition of biofilm formation can be via inhibition of the formation of extracellular polymeric substances (EPS) needed in biofilm formation. This inhibition is possibly through modulating the intracellular c-di-GMP level, as displayed by cranberry water extract on *Vibrio cholerae* [106].

#### 3.4.5. Inhibition of Virulence Factor Production

Phytochemicals like polyphenols, flavonoids and alkaloids have been reported to possess the ability to suppress the production of virulence factors like cholera toxin (CT), haemolysin and proteases (Table 3). For example, in a study by Saha et al. [32], methanol extract of *Typhonium trilobatum* (L.) Schott significantly (*p* < 0.01) inhibited CT production in multidrug-resistant *Vibrio cholerae* (serotypes O1, O139, non-O1 and non-O139), with its production reduced by 76.26%, 48.76% and 29.93% at MIC (8 mg/mL), ½ MIC (4 mg/mL) and ¼ MIC (2 mg/mL), respectively. This was mainly attributed to the ability of the extract to repress the genes, ctxAB, vpsR, rmbA and vpsT, and reduce the expression of CT activator genes, toxT and tcpP. In a similar study, *Centella asiatica* extract displayed the ability to reduce the production of CT in *Vibrio cholerae* strains (El Tor variant, El Tor and Classical). The ctxA gene expression was reduced (i.e., 6.19-, 4.29- and about 10.13- to 0.20-fold, in classical El Tor, and variant strains, respectively) [112].

#### 3.4.6. Inhibition of QS

According to Boyaci et al. [126], QS is an essential signalling system that controls bacterial physiology in response to environmental changes. The system is essential for controlling the synthesis of virulence factors, the formation of biofilms, motility and the change from a motile to a sessile existence in *V. cholerae* [106]. Modulating the QS could potentially serve as a strategy to reduce pathogenicity in *V. cholerae* [126]. Table 3 shows plant compounds, such as terpenoids, flavonoids and phenolic compounds, along with their impact on the QS systems of *V. cholerae*. More importantly, research into anti-QS compounds from medicinal plants that are already used in traditional medicine to treat diarrhoea or cholera can result in the development of novel medications.

#### 3.4.7. Inhibition of Virulence Production

Several studies, including reviews and experiments, have shown the remarkable ability of various plant extracts and isolated phytochemicals to inhibit the production of virulence by different pathogenic bacteria [127–129]. For example, capsaicin, which is present in plants belonging to the *Capsicumin* genus, has been discovered to successfully inhibit the production of CT in different strains of *V. cholerae*, irrespective of their serogroups and biotypes, mostly by reducing the expression of *V. cholerae* toxin genes (e.g., ctxA, tcpA and toxT) [130–132]. Thus, regular consumption of spices like red chilli may be a useful approach to combat the detrimental effects of cholera [133]. In the same way, polyphenols derived from *Haematoxylon brasiletto* extracts were found to inhibit the production of enterotoxin in *V. cholerae* O1 and O139 [134], as well as verotoxin in *Escherichia coli* O157:H7 [135]. In addition, the ethanolic extracts of *Acacia farnesiana* and *Artemisia ludoviciana* were found to also inhibit the production of enterotoxins and adhesion of *V. cholerae* strains (O1 and O139) to cells [136].

## 4. Conclusion

Cholera has become a glaring global health concern due to its elevated rates of morbidity and mortality, particularly in developing and impoverished countries, especially within sub-Saharan region. Cholera is mostly transmitted through the consumption of faecal materials. To manage and prevent cholera infection, it is essential to ensure a continuous supply of clean water and enforce efficient sanitation and hygiene measures. Since 1817, seven cholera pandemics have occurred, originating from Asia and affecting various regions around the world. Cholera outbreaks have occurred sporadically in Zimbabwe since its first occurrence in 1971. Preventing outbreaks entirely is a challenging endeavour due to multiple factors, including migration, inadequacies in water and sanitation infrastructure, inadequate healthcare systems, prolonged and severe droughts, political interference in health matters leading to violations of human rights and the politicisation of water, health care, aid and information. Treating cholera can be difficult in specific circumstances due to the rise of *Vibrio cholerae* strains that are resistant to many drugs, as well as the logistical challenges associated with delivering vaccines. Further research is required to explore other therapeutic options for cholera. Adding natural products—specifically, medicinal plants with potent antibacterial and neutralising properties—to contemporary cholera treatment is critical to improving its efficacy. This is particularly critical in areas where cholera rates are high, and resources are scarce. Certain medicinal plants with significant anticholera properties are considered promising for the treatment and control of cholera. However, the findings of the study should not be regarded as wholly definitive, given the limitations of the methodology used. These limitations may encompass publication bias, restricted access to full texts, geographic and cultural boundaries, among others. Consequently, relying solely on electronic databases could lead to an overestimation of the therapeutic potential of certain plants, while simultaneously underestimating others that are equally significant but less researched. This approach may also overlook some drivers of cholera outbreaks that are just as crucial as those that are more commonly reported. Surveys and observation may serve as valuable additions to electronic database research moving forward.

## Figures and Tables

**Table 1:** Plant species used in Zimbabwe to treat and manage cholera.

Plant species	Local names	Plant parts used and traditional preparation of remedy	Reported antimicrobial activity	Phytochemical analysis	References
*Ziziphus mucronata* Willd.	Muchecheni (shona), Umpasamala (Ndebele)	- Decoction of powdered leaves or roots drunk - Raw or dried stem bark chewed	Exhibited antimicrobial activity with MIC of < 1 mg/mL against a wide range of pathogens	Phenolic acids, alkaloids, flavonoids, terpenoids, quinones, etc.	[81–83, 92]
*Kirkia acuminata* Oliv.	Mubvumira (shona) Umvumile (Ndebele)	- Bark decoction drunk - Powdered bark mixed with food and eaten - Bark infusion drunk	Inhibited *Mycobacterium smegmatis* and *Staphylococcus aureus* at the lowest concentration of 2.5 mg/mL and 1.25 mg/mL.	Fatty acids, flavonols, isocoumarin, lignans, neolignans, nor-carotenoids, phenols, tannins, etc.	[23, 24, 84–87]
*Crossopteryx febrifuga* Benth.	Mukomberwa (shona) Umphokophokwana (Ndebele)	- Bark decoction drunk - Powdered bark mixed with porridge - Powdered bark put into a horn and blown into rectum	Inhibited growth of several bacterial species, with zones of inhibition ranging from 7 to 23 mm	Steroids, flavonoids, terpenoids, anthraquinones and cardiac glycosides, tannins, alkaloids, saponins, etc.	[85, 88]
*Sclerocarya birrea* Hochst.	Mupfura (shona)	- Decoction of crushed bark drunk - Crushed bark mixed with cold water and taken orally or anally - Roots - Leaves	Strong antibacterial activity, with inhibition zones ranging from 19 to 22 mm at 100 mg/mL using various solvents.	Alkaloids, flavonoids, tannins, steroids, triterpenes, glycosides, etc.	[89–91]

**Table 2:** Disruption of cell membrane, synergy or potentiation of commonly used antibiotics and inhibition of protein synthesis and function.

Phytochemical: Plant species	Solvent	Bacteria	Key results and remarks	References
*Disruption of cell membrane*				
Tannins (polyphenols): Gambir (*Uncaria gambir* Roxb.)	Not mentioned	*Enterococcus faecalis*	Molecular docking revealed that tannins had a binding affinity of more than −7.4 kcal·mol^−1^ to the Muramidase B (MurB) enzyme, which is responsible for peptidoglycan biosynthesis. The interaction might inhibit the action of the MurB enzyme, hence preventing bacterial cell wall formation	[34]
Gallotannin-1,2,3,4,5-penta-O-galloyl-*β*-D-glucose (PGG) (tannins)	Not mentioned	*Staphylococcus aureus*	Antibacterial activity was demonstrated by PGG. PGG caused the lipid bilayer to become more fluid, which exposes bacterial membrane damage. There was additional evidence of noncovalent bond interaction between PGG and membrane proteins	[95]
*Sapindus* saponins (Sapindoside A and B)	Water	*Micrococcus luteus*	Synergistic cell membrane attack revealed with Raman spectroscopy showing serious changes in features and constitutions of membrane proteins. Molecular docking also demonstrated Sapindoside A interacting with penicillin-binding protein 2	[96]
Sapindoside A and B (SAB)	Water	*Cutibacterium acnes*	S_AB_ led to the leakage of ions and disturbed the membrane morphology. Spectral changes on cell membrane composition were revealed based on Raman spectroscopy	[97]
Purified flavonoids glycosides from *Graptophyllum glandulosum* Turrill	Methanol, ethanol, butanol	Multidrug-resistant *Vibrio cholerae* strains	Antibacterial activity due to cell lysis and disruption of the cytoplasmic membrane upon membrane permeability was revealed	[29]
Saponins (Panaxydol)	Not mentioned	Propionibacterium	The discharge of cellular components including protein showed cellular and membrane disturbances. Microscopic techniques revealed the disruption of the cell integrity leading to death	[98]
Polyphenolic fraction (composed of catechin and isorhamnetin)	Not mentioned	*V. cholerae* N16961	Morphological alterations disrupted bacterial membrane integrity and an increase in the production of intracellular reactive oxygen species (ROS) were noted. Thus, the basic antibacterial mechanism involved membrane permeabilisation and morphological changes, which might be due to the generation of intracellular ROS	[28]
Flavonoids, quercetin, naringenin and catechin	Not mentioned	*Staphylococcus aureus* strain NCTC 5655	Quercetin increased the registered bacterial cell diameter and enhanced the fluidity of the inner and surface regions of bacterial cell membranes. From the findings, it can be confirmed that cell membrane disorder could be one of the direct modes of antibacterial action of the flavonoids	[99]
Catechin is a plant polyphenol	Not mentioned	*Bacillus subtilis*, *Escherichia coli*	SEM images revealed membrane damage with morphological changes. The inhibitory mechanism of catechin was by oxidative damage through membrane permeabilisation	[100]

*Synergy or potentiation of commonly used antibiotics*				
Saponin-rich fraction	Ethanol	*Escherichia coli*, *Pseudomonas aeruginosa*	Potentiated the activity of aminoglycosides and fluoroquinolones. For example, enhanced the effect of gentamicin against both bacteria and also when combined with norfloxacin against *P. aeruginosa*	[101]
Friedelane-type triterpenoid and phytosterols (*β*-sitosterol)	Methanol, ethyl acetate	*E. coli* and *P. aeruginosa*	Potentiation of amoxicillin was shown. In addition, the compounds demonstrated the ability to block the bacterial efflux pumps at ½ MIC and ¼ MIC against *E. coli* and *P. aeruginosa*	[35]
Polyphenol (−)-epigallocatechin-3-gallate (EGCG)	Not mentioned	Multidrug-resistant *V. cholerae*	The combination of EGCG and tetracycline was greater than either treatment alone, suggesting a positive synergistic potential of the two. SEM showed membrane-disrupting permeability as mode of action of EGCG	[36]
Coffee beans and coffee by-products	Water	Drug-resistant *V. cholerae*	The checkerboard assay revealed that the combination of the extract and tetracycline was greater than either treatment alone. SEM revealed antibacterial mechanism was via disrupting membrane permeability	[102]

*Inhibition of protein synthesis and function*				
Flavonoid [morin (3,5,7,2′,4′-pentahydroxyflavone)]	Not mentioned	*V. cholerae*	Morin was able to bind to major cell division protein of *V. cholerae* (VcFtsA) with a Kd of 4.68 ± 0.4 *μ*M, inhibiting the protein's polymerisation by 72% ± 7% at 25 *μ*M concentration and in turn affected the cell division in *V. cholerae*. Morin also affected VcFtsA's ATPase activity, recording ∼80% reduction at 20 *μ*M concentration	[27]
Tannins (polyphenols): Gambir (*U. gambir* Roxb.)	Not mentioned	*E. faecalis*	Molecular docking revealed that tannins had a binding affinity of more than −7.4 kcal·mol^−1^ to the Muramidase B (MurB) enzyme, which is responsible for peptidoglycan biosynthesis. The interaction might inhibit the action of the MurB enzyme, hence preventing bacterial cell wall formation	[34]
Catechin (flavonoid): Gambir (*U. gambir* Roxb.)	Not mentioned	Not mentioned	Catechin exhibited a binding affinity of over −7 kcal/mol towards the MurB enzyme, which may have implications for both cell wall and peptidoglycan biosynthesis	[34]
*β*-sitosterol (phytosterol): *Ocimum basilicum* L. leaves	Methanol	*E. faecalis* ATCC 29212, *Streptococcus mutans* ATCC 25175, *Streptococcus sanguinis* ATCC 10566	*β*-sitosterol exhibited the potential to inhibit the biosynthesis of peptidoglycan and prevent bacteria cell wall formation by inhibiting MurA via docking simulation	[103]

**Table 3:** Inhibition of biofilm formation, quorum sensing and virulence factor production.

Phytochemical: Plant species	Solvent	Bacteria	Key results and remarks	References
*Inhibition of biofilm formation*				
*Sapindus* saponins (from seed pericarps), including oleic acids	Methanol	Methicillin-resistant *Staphylococcus aureus*	Exhibited 82% of biofilm inhibition at 250 mg/mL. Reduction in slime, cell surface hydrophobicity, autoaggregation, extracellular polysaccharides substance and extracellular DNA synthesis were demonstrated. The ability of the extract to interfere with the expression of adhesion genes associated with biofilm formation (e.g., icaA, icaD, fnbA, fnbB, clfA, cna and altA) was confirmed. These results suggest that extract can be used as a promising therapeutic agent against biofilm-associated infections	[104]
Flavonoids: *Nephelium lappaceum* L. cv. Rong Riaan peel	Water	*Vibrio cholerae*	A reduction in biofilm formation was demonstrated. A significant downregulation of genes involved in biofilm formation (*vasal*, *vpsR*, *bap1* and *rbmA*) was observed	[25]
*Typhonium trilobatum* (L.) Schott extract	Methanol	Multidrug-resistant *V. cholerae* (serotypes O1, O139, non-O1 and non-O139)	The extract showed significant (*P* < 0.05) inhibitory activity on the formation of biofilm, with 81.3%, 75.8% and 69.6% of inhibition at MIC, ½ MIC and ¼ MIC, respectively. Showed also inhibitory activity on the formation of extracellular polymeric substances (EPS) formation, with 89.41%, and 99.26% of inhibition of EPS protein and EPS carbohydrate at MIC, respectively	[32]
Quercetin and naringenin	Not mentioned	*Chromobacterium violaceum*, *V. cholerae*	Though the bacteria were not killed, a two-fold reduction in the expression of biofilm-associated genes (*gbpA*, *vpsA*, *rbmA* and *mbaA*) was observed. In addition, the phytomolecules had antiadhesion and anti-invasion properties	[26]
Quercetin, morin, vanillic acid and protocatechuic	Not mentioned	*V. parahaemolyticus* (Vp124 and Vp320)	Biofilms biomass was reduced by 63.22%–92.68%. In addition, quercetin and morin inhibited the motility of both strains by 15.86%–23.64% (Vp124) and 24.28%–40.71% (Vp320)	[33]
Flavonoid derivatives (6-aminoflavone, 3,2-dihydroxyflavone and 2,2-dihydroxy-4-methoxybenzophenone)	Not mentioned	*Vibrio parahaemolyticus*, *V. harveyi, Staphylococcus aureus* and *Salmonella typhimurium*	Disrupted preformed biofilms and suppressed virulence properties including motilities, cell hydrophobicity and aggregation. The flavonoids also interfered with the metabolic activity, cell division and membrane permeability to exert antibiofilm and antibacterial activities.	[105]
Cranberry extract	Water	*V. cholerae*	The extract significantly inhibited biofilm formation by reducing the biofilm matrix production and secretion. This was possibly through modulating the intracellular c-di-GMP level	[106]
*Centella asiatica*, *Elephantopus scaber*, *Camellia sinensis* and *Holarrhena antidysenterica* extracts	Methanol	Toxigenic *V. cholerae*	The extracts significantly inhibited biofilm formation by approximately 75%, 76%, 78% and 55%, respectively. The genes (aphA or aphB) responsible for biofilm formation were downregulated by H. antidysenterica, E. scaber and *C. asiatica* extracts	[107]
Resveratrol	Not mentioned	*V. cholerae*	Subinhibitory concentrations of the compound significantly inhibited biofilm formation. Docking analysis showed that resveratrol could target *Aph*B, resulting in suppression of *V. cholerae* virulence gene expression	[108]

*Inhibition of quorum sensing*				
Flavonoids: Nephelium lappaceum L. cv. Rong Rian peel	Water	*V. cholerae*	A significant downregulation of genes involved in *V. cholerae* quorum sensing regulator (*luxO*) was observed	[25]
*Alpinia nigra* leaf extract	Ethyl acetate	*Pseudomonas aeruginosa.*	The *Alpinia nigra* leaf extract (L-EtAc) displayed inhibiting potential of QS signalling. L-EtAc played a vital role in antibiofilm activity towards *P. aeruginosa*	[109]
Catechin and gallic acid (from *Pelargonium* × *hortorum root extract*)		*P. aeruginosa*	The combination of the isolated plant compounds downregulated *las*I and *las*R gene expression responsible for biofilm formation in *P. aeruginosa.* The potential biofilm and quorum sensing inhibitory activity of the catechin and gallic acid combination was demonstrated	[110]
Betulin and epicatechin gallate (from *Acacia nilotica* extract)	*n*-Hexane, chloroform and ethyl acetate	Gram-negative pathogens	The phytochemicals, displayed ability to interact with biofilm proteins PilY1 and PilT, preventing biofilm formation. The findings suggest that the phytochemicals could be exploited as an anti-QS agent against Gram-negative pathogens	[111]
*Piper betle* L. leaf extract	Ethanol	*Vibrio harveyi*	The extract inhibited quorum sensing in *Vibrio harveyi,* as well as biofilm formation	[30]

*Inhibition of virulence factor production*				
*T. trilobatum* (L.) Schott extract	Methanol	Multidrug-resistant *V. cholerae* (serotypes O1, O139, non-O1 and non-O139)	The extract significantly (*p* < 0.01) inhibited cholera toxin (CT) production with its production reduced by 76.26%, 48.76% and 29.93 at MIC (8 mg/mL), ½ MIC (4 mg/mL) and ¼ MIC (2 mg/mL), respectively. The genes *ctxAB*, *vpsR*, *rmbA* and *vpsT* were repressed. In addition, the expression of cholera toxin activator genes, *toxT* and *tcpP*, was reduced.	[32]
*Centella asiatica* extract	Not mentioned	*V. cholerae* (El Tor variant, El Tor, Classical)	A reduction of 70%, 89% and 93% CT produced by classical, El Tor and variant, respectively. The ctxA gene expression was reduced (i.e., 6.19-, 4.29- and about 10.13- to 10.20-fold, in classical El Tor, and variant strains, respectively)	[112]
Hydroquinones (2,3-dimethylhydroquinone and 2,5-ditert-butylhydroquinone)	Not mentioned	*Vibrio parahaemolyticus*	The expression of virulence-related genes was repressed and production of protease, indole as well as motility phenotypes were inhibited. 2,3-Dimethylhydroquinone decreased fimbriae production	[31]

## Data Availability

There are no data available for this review manuscript.
